# Novel perspective on immune cell regulation in gastrointestinal inflammation: the role of extracellular vesicles and therapeutic prospects

**DOI:** 10.3389/ebm.2026.11114

**Published:** 2026-07-17

**Authors:** Jianshu Wang, Junxuan Xu, Zilu Cui, Jing Wu

**Affiliations:** Department of Gastroenterology, Beijing Friendship Hospital, Capital Medical University, State Key Laboratory of Digestive Health, National Clinical Research Center for Digestive Disease, Beijing Digestive Disease Center, Beijing, China

**Keywords:** cellular crosstalk, extracellular vesicles, gastrointestinal inflammation, immune cells, nanomedicine

## Abstract

Gastrointestinal inflammation is an inflammatory disease arising from immune imbalance in any segment of the digestive tract, triggered by various factors. Immune cells play important roles in both the onset and resolution of gastrointestinal inflammation. With the recent extensive research on extracellular vesicles, the mechanism by which immune cells regulate gastrointestinal inflammation through extracellular vesicles has gradually gained recognition within the scientific community. Extracellular vesicles derived from immune cells can communicate with other immune cells in the digestive tract and directly regulate digestive tract epithelial cells. Furthermore, with advances in biological nanotechnology, immune cell-derived extracellular vesicles may be used to treat inflammatory gastrointestinal diseases. This review focuses on delineating the role of immune cell-derived extracellular vesicles in gastrointestinal inflammation and exploring their potential applications in treating these inflammatory diseases.

## Impact statement

The incidence and prevalence of gastrointestinal inflammation are rising annually, and the treatment options are limited, which have brought huge burdens to both society and individuals. Immune cells play crucial roles in the pathophysiology of gastrointestinal inflammation. With the emergence of new, groundbreaking evidence in recent years, there is an urgent need to summarize the roles of immune cells in gastrointestinal inflammation and to identify future research directions. This review approaches gastrointestinal inflammation from a novel perspective — immune cell-derived extracellular vesicles — to establish their central roles in the onset and progression of the disease. Crucially, we have mapped the functional network of immune cell-derived extracellular vesicles in gastrointestinal inflammation, which visually illustrates the roles of various immune cells and their crosstalk with other cells, thereby providing insights for future research. Furthermore, we highlight promising modification strategies for extracellular vesicles and their therapeutic potential for gastrointestinal inflammation. Notably, to facilitate a better understanding of currently known modification strategies, we have innovatively classified these approaches in a simple, intuitive way based on their fundamental principles. This work comprehensively summarizes and thoroughly discusses the emerging mechanisms of immune cell derived extracellular vesicles in gastrointestinal inflammation, as well as the latest advances in modifying these vesicles to enhance therapeutic efficacy. We not only provide new insights into the mechanisms by which immune cells contribute to gastrointestinal inflammation but also offer ideas for developing novel therapeutics to treat this complex disease, thereby advancing the field.

## Introduction

Gastrointestinal inflammation is a major category of digestive system diseases. Such inflammatory conditions present a broad spectrum of complex symptoms, and their chronic persistence may trigger tumor development [1, 2]. Furthermore, both the incidence and prevalence of gastrointestinal inflammation are rising annually, which poses substantial challenges to healthcare systems [3–7]. Existing research suggests that immune dysregulation plays an important role in the development of these inflammatory diseases, and the key functions and regulatory mechanisms of immune cells involved in the pathophysiological process of gastrointestinal inflammation have been studied [8–11].

The mechanisms by which immune cells exert their effects in gastrointestinal inflammation largely depend on extracellular vesicles (EVs). EVs are lipid bilayer membrane vesicles released by different cells [12]. Although the specific mechanisms of EV formation are not fully understood, their functional effects in many physiological and pathological conditions have been extensively studied. These vesicles contain multiple functional units, primarily comprising proteins, nucleic acids, and lipids [12]. Through these components, EVs play central roles in intercellular communication. These vesicles transport and deliver proteins or nucleic acids to receptor cells, regulating various pathological and physiological processes, including inflammatory responses [13]. EVs can also serve as effective drug carriers for disease treatment [14]. Beyond protecting drug stability, the surface of EVs contains various adhesion molecules, exhibiting strong affinity for cell membranes [15]. In addition, EVs effectively avoid immune rejection responses [15]. Given their endogenous nature and multifunctional capabilities, investigating the role of immune cell-derived EVs in gastrointestinal inflammation not only sheds further light on the mechanisms underlying such inflammatory conditions but also offers novel insights into therapeutic management.

In this review, we described the roles of immune cell-derived EVs in gastrointestinal inflammation and discussed approaches to modifying EVs and their therapeutic potential as nanomedicines.

## Characteristics of EVs

EVs are double-layered lipid-enveloped membrane vesicles, containing lipids, proteins, nucleic acids (DNA, mRNA, miRNA, and LncRNA), and other bioactive substances [16]. These vesicles are widely distributed in tissues and bodily fluids, playing important roles in intercellular regulation during physiological and pathological processes [17]. Based on their origin and biogenesis, EVs can be classified into different types, primarily including exosomes, ectosomes, and others (migrasomes, apoptotic EVs). Although the scientific community does not yet fully comprehend the biogenesis and release processes of EVs, certain key factors or mechanisms have been reported [18, 19]. In brief, exosomes are first generated by endosomal inward budding, followed by fusion of endosomal compartments with the plasma membrane, thereby releasing exosomes into the extracellular space. The biogenesis of ectosomes is comparatively straightforward. They arise from direct outward budding of the plasma membrane. Other EV subtypes, such as migrasomes, are generated during cell migration, and apoptotic cells undergoing fragmentation produce apoptotic EVs.

The most recent edition of the Minimal Information for Studies of Extracellular Vesicles (MISEV) proposes straightforward and efficient classification methods that address the complexity of EV types and the uncertainties in their biogenesis [16]. For example, using a particle size of 200 nm as the dividing line, EVs are divided into large EVs (>200 nm) and small EVs (<200 nm). Another classification criterion relies on defined density ranges. Using this method, EVs are categorized into low-density, medium-density, and high-density subtypes. Furthermore, EVs can be classified by their biochemical composition, specifically the presence or absence of particular molecules such as proteins. Notably, certain signature proteins can indicate the intracellular origin of EVs. Finally, classification can be performed based on the cellular origin of EVs and the conditions under which they are generated. This approach places greater emphasis on distinct biogenetic pathways, including energy dependence (or lack thereof), as well as the functional status of parent cells under stress or cell death.

It has been known that EVs play significant roles in intercellular communication. Cells communicate with each other by secreting signaling molecules, which they can package within EVs to evade rapid degradation and escape immune surveillance, thereby enabling both short- and long-distance intercellular communication [20]. The functional effects of EVs under numerous pathological and physiological conditions have been studied. EV-mediated intercellular communication is important for maintaining normal physiological functions, whilst abnormal EV signaling is associated with diverse disease states, including neurological disorders, cardiovascular diseases, renal diseases, endocrine disorders, viral infections, immune diseases, and various cancers [17, 21, 22]. EVs and their protein, lipid, and nucleic acid cargos are potential biomarkers and therapeutic targets [17, 23]. Moreover, EVs are gradually being explored as carriers for drug delivery [17, 23].

## Roles of immune cell-derived EVs in gastrointestinal inflammation

**FIGURE 1:**
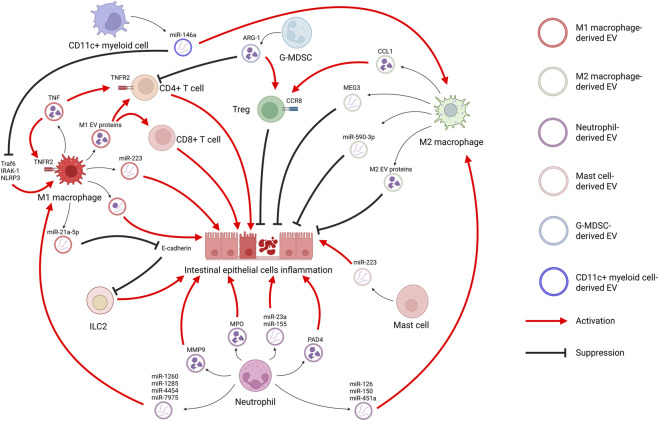
Roles of immune cell EVs in colitis. This figure shows that M1 macrophages, M2 macrophages, neutrophils, mast cells, G-MDSCs, and CD11c+ myeloid cells can participate in the biological processes of colitis through their derived EVs and EV contents (Proteins and RNAs). M1 macrophage-derived EVs exert pro-inflammatory effects via delivery of miR-223, miR-21a-5p, TNF, and related proteins. Neutrophil-derived EVs exert pro-inflammatory effects via delivery of MMP-9, MPO, PAD4, miR-23a, miR-155, miR-1260, miR-1285, miR-4454 and miR-7975, and anti-inflammatory effects via delivery of miR-126, miR-150 and miR-451a. Mast cell-derived EVs exert pro-inflammatory effects via delivery of miR-223. M2 macrophage-derived EVs exert anti-inflammatory effects via delivery of miR-590-3p, LncRNA MEG3, CCL1, and related proteins. G-MDSC-derived EVs exert anti-inflammatory effects via delivery of Arg-1. CD11c^+^ myeloid cell-derived EVs exert anti-inflammatory effects via delivery of miR-146a. These EVs can act directly on intestinal epithelial cells or function indirectly by targeting other immune cells and modulating their activities. All the above processes constitute a complex immune regulatory network. Created with BioRender.com.

**FIGURE 2:**
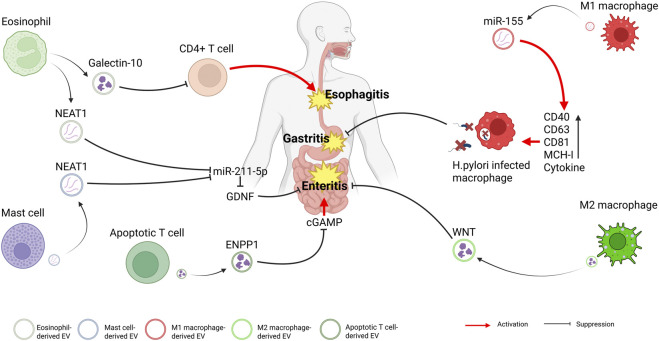
Roles of immune cell EVs in other gastrointestinal inflammation. This figure shows that Eosinophil, mast cell, M1 macrophage, M2 macrophage, and Apoptotic T cell can participate in the biological process of gastrointestinal inflammation other than colitis through their derived EVs and EV contents (Proteins and RNAs). Eosinophil-derived EVs exert anti-inflammatory effects via delivery of galectin-10 and NEAT1. Mast cell-derived EVs exert anti-inflammatory effects via delivery of NEAT1. M1 macrophage-derived EVs exert anti-inflammatory effects via delivery of miR-155. M2 macrophage-derived EVs exert anti-inflammatory effects via delivery of WNT. T cell-derived EVs exert anti-inflammatory effects via delivery of ENPP1. These EVs can act directly on gastrointestinal epithelial cells or function indirectly by targeting other immune cells and modulating their activities. The anti-inflammatory effects of M1 macrophages are mediated indirectly via their secreted EVs, which enhance macrophage bactericidal activity. Created with BioRender.com.

Cellular communication is vital to the homeostasis of the digestive system [24, 25]. EVs derived from immune cells have been shown to play significant roles in the mechanisms of inflammatory exacerbation and resolution by interacting with gastrointestinal epithelial cells and other cell types. In the following section, we discuss the roles of immune cell-derived EVs in gastrointestinal inflammation (Table 1). In this field of research, most studies have focused on colitis. Consequently, we shall discuss the roles of immune cell-derived EVs in colitis (Figure 1) and other gastrointestinal inflammation (Figure 2) separately.

**TABLE 1:** Roles of immune cell EVs in gastrointestinal inflammation.

Disease	Source of EVs	Key cargos of EVs	Target site	Main effects	References
Inflammatory bowel disease	M1 macrophages	Unclear	TLR4 signaling pathway	Promote mouse colitis progression by activating the TLR4 signaling pathway	[26]
​	​	miR-223	TMIGD1	Induce intestinal barrier dysfunction through the inhibition of TMIGD1	[27]
​	​	TNF	NF-κB signaling pathway	Protect CD4^+^ T cells from activation-induced cell death via the TNF/TNFR2/NF-κB axis	[28]
​	​	miR-21a-5p	E-cadherin	Reduce E-cadherin and thus activate ILC2s through KLRG1/GATA-3 axis	[29]
​	​	TNF	NF-κB signaling pathway	Trigger glycolytic activation and inflammation response in macrophages via the TNF/TNFR2/NF-κB axis	[30]
​	​	M1 EV proteins	T Lymphocytes	Promote viability, proliferation, and activation of T lymphocytes	[31]
​	M2 macrophages	CCL1	CCR8	Protect epithelial cells through the CCL1/CCR8 axis	[32]
​	​	miR-590-3p	LATS1/YAP/β-catenin axis	Promote epithelial regeneration through the LATS1/YAP/β-catenin axis	[33]
​	​	LncRNA MEG3	miR-20b-5p	Enhance cell viability and reduce inflammatory responses via the miR-20b-5p/CREB1 axis	[34]
​	​	M2 EV proteins	Unclear	Protect the tight junction structure and barrier integrity of epithelial cells	[31]
​	Neutrophils (proinflammatory)	MMP-9	DSG-2	Induce the division of DSG-2, thereby leading to the loss of cadherins and disrupting the adhesion between IECs	[35]
​	​	MPO	Unclear	Injury to IEC migration and proliferation, leads to inhibition of IEC wound healing	[36]
​	​	PAD4	CKMT1	Induce the citrullination of CKMT1, thus disrupting mitochondrial homeostasis and leading to apoptosis of IECs	[37]
​	​	miR-23a and miR-155	DSBs	Promote the accumulation of DSBs, leading to impaired colon healing and genomic instability	[38]
​	​	miR-1260, miR-1285, miR-4454, and miR-7975	Macrophage polarization	Enhance the polarization of proinflammatory macrophages	[39]
​	Neutrophils (anti-inflammatory)	miR-126, miR-150, and miR-451a	Macrophage polarization	Enhance the polarization of anti-inflammatory macrophages	[39]
​	Mast cells	miR-223	CLDN8	Inhibit the expression of CLDN8, thus disrupting intestinal barrier function	[40]
​	MDSCs	Arg-1	T Lymphocytes	Inhibit the proliferation of Th1 cells and promote the proliferation of Tregs	[41]
​	CD11c+ myeloid cells	miR-146a	Traf6/IRAK-1/NLRP3 axis	Regulate macrophage polarization and reduce intestinal inflammatory response through the Traf6/IRAK-1/NLRP3 axis	[42]
Eosinophilic esophagitis	Eosinophils	Galectin-10	T Lymphocytes	Inhibit the function of T cells	[43]
Functional dyspepsia	Eosinophils and mast cells	LncRNA NEAT1	miR-211-5p	Promote duodenal mucosa integrity by miR-211-5p/GDNF axis	[44]
Radiation enteritis	Apoptotic T cells	ENPP1	cGAMP	Alleviate radiation enteritis by hydrolyzing cGAMP and inhibit cGAS-STING pathway	[45]
​	Macrophages	WNTs	Unclear	Rescue intestinal stem cells after radiation injury	[46]
*H. pylori* gastritis	Macrophages	miR-155	Unclear	Promote the expression of inflammatory cytokines, thus leading to the clearance of *H. pylori*	[47]

*EVs*, extracellular vesicles; *MDSCs*, myeloid-derived suppressor cells, *TLR4* toll-like receptor 4, *TMIGD1* transmembrane and immunoglobulin domain containing 1, *TNF*, tumor necrosis factor, *TNFR2* tumor necrosis factor receptor 2, *NF-κB*, nuclear factor kappa B, *ILC2s* type 2 innate lymphoid cells, *KLRG1* killer cell lectin like receptor G1, *GATA-3*, GATA-binding protein 3, *LATS1* large tumor suppressor kinase 1, *YAP*, yes-associated protein, *MEG3* maternally expressed gene 3, *CREB1* cAMP, responsive element binding protein 1, *CCL1* C-C motif chemokine ligand 1, *CCR8* C-C motif chemokine receptor 8, *DSG-2*, desmoglein-2, *MMP9* matrix metalloproteinase 9, *IEC*, intestinal epithelial cell; *MPO*, myeloperoxidase; *DSBs*, double-strand breaks, *CKMT1* mitochondrial creatine kinase 1, *PAD4* peptidyl arginine deiminase 4, *CLDN8* claudin 8, *Th1* type 1 helper T, *Tregs* regulatory T cells, *Arg-1*, arginase-1, *NEAT1* nuclear-enriched abundant transcript 1, *GDNF*, glial cell line-derived neurotrophic factor; *WNTs*, wingless-related integration site; *cGAMP*, 2′3′cyclic GMP-AMP, *cGAS*, cyclic GMP-AMP, synthase; *STING*, stimulator of interferon genes, *ENPP1* ectonucleotide pyrophosphatase phosphodiesterase 1, *Traf6* TNF, receptor associated factor 6; *IRAK-1*, interleukin 1 receptor associated kinase 1, *NLRP3* NLR, family pyrin domain containing 3, *H. pylori helicobacter pylori*.

### Roles of immune cell-derived EVs in colitis

#### Macrophages

Regarding the effects of immune cell-derived EVs on colitis, macrophages are currently the most extensively studied cell type. In fact, the role of macrophage-derived EVs in colitis represents a current research focus. Depending on the activation pathway, macrophages are polarized into M1 (classical activation) or M2 (alternative activation) macrophages. Classical activation induces M1 macrophages to produce pro-inflammatory cytokines, whereas alternative activation causes M2 macrophages to initiate anti-inflammatory responses.

Compared to EVs derived from unpolarized M0 macrophages, M1 macrophage-derived EVs act as pro-inflammatory mediators that significantly exacerbate colitis and tissue inflammation in mice, activate TLR4 signaling, and disrupt the mucosal barrier [26]. This effect can be demonstrated through the antagonistic action of Resatorvid [26]. Further research has revealed key pro-inflammatory molecules within these vesicles. In Chang’s study, LPS-induced macrophage EVs delivered substantial amounts of miRNAs to intestinal epithelial cells, wherein miRNA-223 significantly suppressed TMIGD1 function, thereby inducing intestinal barrier dysfunction and promoting colitis progression [27].

As nanoscale lipid vesicles, EVs mediate the crosstalk between various immune cells. For instance, EVs derived from intestinal CD14^+^ macrophages can protect CD4^+^ T cells from activation-induced cell death [28]. The mechanisms involve binding of membrane-bound TNF to TNFR2, followed by activation of the NF-κB signaling pathway [28]. This process directly leads to the sustained activation of pro-inflammatory Th1 and Th17 cells, playing an important role in the pathogenesis of Crohn’s disease [28]. Interestingly, the patterns of crosstalk between immune cells via EVs are diverse. Besides the classical direct crosstalk mode mentioned above, immune cells can also use other cells as ' transit stations ' to crosstalk indirectly. It has been reported that M1 macrophage-derived EVs can exacerbate inflammatory bowel disease by reducing E-cadherin and activating group 2 innate lymphoid cells via their cargo miRNA-21a-5p [29]. Specifically, miRNA-21a-5p first targets intestinal epithelial cells and reduces E-cadherin expression [29]. This reduction further promotes excessive activation of group 2 innate lymphoid cells via the KLRG1/GATA-3 axis, thereby intensifying intestinal inflammatory responses [29]. Additionally, immune cells may exert autocrine or paracrine crosstalk via EVs. Research by Zeng et al. revealed that, compared with the control group, patients with Crohn’s disease exhibit marked intestinal inflammatory responses, manifesting as abnormal glycolytic activation of CD14^+^ intestinal macrophages [30]. Mechanistically, macrophage-derived EVs bear membrane-bound TNF [30]. By binding to TNFR2 on the macrophage plasma membrane, this surface TNF activates TNF–NF-κB-dependent autocrine and paracrine signaling pathways. It subsequently drives glycolytic reprogramming and initiates a sequential inflammatory signaling cascade [30].

M2 macrophages and their EVs usually exert anti-inflammatory effects in colitis, promoting tissue repair and maintaining intestinal barrier integrity. Yang et al. explored the anti-inflammatory effects of EVs derived from various M2 macrophage subtypes (M2a, M2b, M2c, and M2d). They found that M2b macrophage EVs exhibited a more pronounced protective effect on DSS-induced inflamed colonic epithelial cells [32]. When focusing on the key effector miRNAs of M2 macrophage-derived EVs, unlike M1 macrophage EVs, which contain the pro-inflammatory miRNA-223, EVs from M2 macrophages are significantly enriched in the anti-inflammatory miRNA-590-3p [33]. This miRNA alleviates inflammation and mucosal damage while promoting the repair and proliferation of intestinal epithelial cells [33]. Long non-coding RNA (LncRNA) regulates gene expression and a variety of molecular pathways involved in the pathophysiology of diseases [48]. M2 macrophages can also deliver LncRNA MEG3 to intestinal epithelial cells via EVs [34]. Through the miRNA-20b-5p/CREB1 axis, LncRNA MEG3 enhances epithelial cell viability and alleviates colonic inflammatory responses [34].

These studies have investigated the roles of EVs derived from M1 or M2 macrophages in colitis. However, the cargos of EVs focused on these studies were mostly limited to various RNAs. In fact, the primary cargos within EVs include not only RNAs but also proteins and lipids. Proteins, in particular, as the ultimate effectors performing various functional roles in cells, play significant roles in promoting or alleviating inflammation. Liu et al. elucidated the roles of protein cargos within M1 and M2 macrophage EVs during intestinal inflammation [31]. Generally, enriched proteins in M1 macrophage EVs induce M1 polarization and participate in pro-inflammatory pathways, as evidenced by their promotion of T lymphocyte vitality, proliferation, and activation [31]. The enriched proteins in M2 macrophage EVs induce M2 polarization and engage in immune regulation and tissue remodeling, as evidenced by their effective protection of the tight junction structure and barrier integrity of intestinal epithelial cells, thereby alleviating colitis [31]. This study depicted the comprehensive landscape of protein cargos in macrophage-derived EVs and emphasized their crucial role in pathophysiological processes. However, the study’s exploration of the mechanism by which key protein cargos exert their effects after delivery to intestinal epithelial cells was relatively superficial. Additionally, the macrophages used in this study were derived from mice rather than humans, which cannot be considered fully equivalent to human macrophages. Therefore, its interpretation of the roles of macrophages in human intestinal inflammation is limited. In summary, the role of protein cargos within macrophage EVs in gastrointestinal inflammation needs further investigation.

#### Neutrophils

In addition to macrophage-derived EVs, neutrophil-derived EVs play significant roles in the pathogenesis and progression of colitis and often exacerbate inflammation. As major myeloid leukocytes of the innate immune system, neutrophils play a central role in host defense against invading pathogens [49]. These cells are also closely involved in numerous pathological states, ranging from acute to chronic inflammation [50]. In the inflammatory microenvironment, neutrophils undergo a series of pathophysiological processes, including recruitment, migration, and activation [49, 51]. After inflammation resolves, neutrophils undergo reverse migration or apoptosis [49, 51]. Neutrophil transepithelial migration (TEM) is a hallmark of inflammatory mucosal diseases and correlates with epithelial damage [52]. During TEM, neutrophil EVs deposit onto intestinal epithelial cells, leading to the loss of cadherins [35]. The key factor matrix metalloproteinase 9 induces cleavage of desmoglein-2, thereby disrupting the epithelial barrier, promoting epithelial damage, and enhancing neutrophil recruitment [35]. Myeloperoxidase (MPO), highly expressed in neutrophil granules, serves to eliminate bacteria [53]. However, Slater et al. discovered that during intestinal inflammation, MPO is transported to the neutrophil surface and subsequently delivered to intestinal epithelial cells through EVs, leading to a marked inhibition of intestinal mucosal epithelial repairment [36]. The mechanism likely involves disrupted actin dynamics, impaired cell motility, and cell cycle arrest, any of which may cause impaired intestinal epithelial cell migration and proliferation [36]. Moreover, Wang et al. found that neutrophils can secrete EVs carrying PAD4 that enter intestinal epithelial cells, thereby inducing CKMT1 citrullination [37]. This process reduced CKMT1 protein stability via the autophagy pathway, thereby disrupting mitochondrial homeostasis and inducing apoptosis in intestinal epithelial cells [37]. This process ultimately led to impaired intestinal barrier integrity and aggravated mucosal inflammation in inflammatory bowel disease [37].

The three aforementioned studies have identified key enzymes responsible for the pro-inflammatory effects of neutrophil-derived EVs. Similar to macrophage-derived EVs, neutrophil-derived EVs can also exert biological functions by delivering critical miRNAs. Butin-Israeli et al. explored the RNA cargo of neutrophil-derived EVs during intestinal inflammation and identified that the pro-inflammatory miRNAs (miRNA-23a and miRNA-155) promote the accumulation of double-strand breaks [38]. This effect in injured epithelium resulted in impaired colonic healing and genomic instability [38]. Conversely, targeted inhibition of miRNA-23a and miRNA-155 reduced neutrophil-mediated harm and enhanced the tissue healing response [38]. It is worth noting that although most EVs derived from neutrophils exhibit pro-inflammatory effects, there are also neutrophil EVs that mainly exhibit anti-inflammatory effects. Moreover, the broader category of neutrophil EVs also includes neutrophil-derived tail, a special type of EV generated during neutrophil migration towards the inflammation sites [54]. Research by Youn et al. on these two special neutrophil EVs revealed that neutrophil-derived trails deliver pro-inflammatory miRNAs, such as miRNA-1260, miRNA-1285, miRNA-4454, and miRNA-7975, thereby enhancing the pro-inflammatory polarization of macrophages [39]. In contrast, neutrophil microvesicles exhibiting anti-inflammatory effects contain anti-inflammatory miRNAs, such as miRNA-126, miRNA-150, and miRNA-451a, which induce the polarization of anti-inflammatory macrophages [39].

Existing studies predominantly regard intestinal epithelial cells as the primary target cells of neutrophil-derived EVs. By contrast, limited research has explored how these vesicles regulate other immune cells. In fact, neutrophils interact closely with various immune cells, including monocytes, macrophages, B cells, and T cells. They are capable of regulating cell differentiation, polarization, and maturation [55–57]. Future investigations should address this knowledge gap, particularly by exploring EV-mediated crosstalk between neutrophils and other immune cells, as well as its impact on colitis. Furthermore, accumulating evidence demonstrates that neutrophils play indispensable roles in tissue injury and repair, a property that is closely associated with neutrophil heterogeneity [58]. Two mainstream hypotheses explain this heterogeneity. One view states that neutrophils possess strong phenotypic plasticity and can remodel their phenotypes in response to external stimuli [59, 60]. The other hypothesis proposes that distinct neutrophil subsets exist under both physiological and pathological conditions [59, 60]. Pro-inflammatory and anti-inflammatory neutrophil subsets may induce polarization of different macrophage subsets, thereby leading to divergent immune outcomes [59, 60]. Accordingly, like macrophages, neutrophils play dual roles in gastrointestinal inflammation. Their complex functions require comprehensive evaluation and further in-depth exploration.

#### Other cells

In addition to macrophages and neutrophils, EVs derived from other immune cells also exert vital effects throughout the initiation and progression of colitis. Mast cells are immune cells that develop from hematopoietic progenitor cells in the bone marrow [61, 62]. These cells actively participate in the pathogenesis of intestinal inflammation. The activation of mast cells in the inflammatory intestine leads to changes such as the recruitment of other inflammatory cells, alterations in barrier function, and tissue remodeling. Research by Li et al. revealed that miRNA-223 within mast cell-derived EVs suppresses CLDN8 expression in intestinal epithelial cells, thereby damaging intestinal barrier function and exacerbating colonic inflammation [40]. Conversely, the application of miRNA-223 inhibitors can significantly reverse the inhibitory effect on CLDN8 expression [40]. Beyond their pro-inflammatory activities, immune cell-derived EVs have also been reported to exert anti-inflammatory effects in colitis. Such protective functions are mediated by EVs and involve cellular crosstalk among immune cells. Myeloid-derived suppressor cells (MDSCs) are widely recognized as a heterogeneous group of immature bone marrow cells that suppress immune responses and participate in the pathophysiology of inflammation, tumors, and pathogen infections [63]. MDSCs are categorized into M-MDSCs and PMN-MDSCs, with the latter exhibiting a granulocyte morphology and thus termed granulocyte-derived MDSCs (G-MDSCs). Wang et al. found that EVs derived from G-MDSCs can enhance mice’s resistance to colitis, as evidenced by a lower disease activity index and reduced inflammatory cell infiltration [41]. The vesicles suppress Th1 cell proliferation and inflammatory cytokine release while promoting the proportion of regulatory T cells (Tregs). These effects are largely mediated by the EV cargo arginase-1 [41]. In addition, CD11c+ myeloid cells may also alleviate intestinal inflammation by cross-talking with other immune cells. Bauer et al. demonstrated that EVs derived from CD11c+ myeloid cells can deliver the anti-inflammatory miRNA-146a to macrophages via a Rab27a-dependent mechanism [42]. Targeting Traf6, IRAK-1, and NLRP3, these vesicles modulate macrophage polarization and reduce intestinal inflammatory responses [42].

### Roles of immune cell-derived EVs in other gastrointestinal inflammations

Beyond colitis, immune cells and their EVs also play significant roles in other forms of gastrointestinal inflammation. Most existing studies have revealed their predominant anti-inflammatory effects. Eosinophilic esophagitis is a T-cell-driven allergic disease characterized by eosinophilic infiltration of the esophagus [64]. Eosinophils can secrete cytokines, chemokines, and cationic proteins, transporting and releasing these mediators to contribute to inflammation and other immune responses [65]. Current research indicates that, in addition to these functions, eosinophils can also generate EVs, and this process is enhanced under inflammatory stimulation [66]. Albinsson et al. discovered that eosinophils suppress T-cell function by releasing galectin-10, despite the lack of direct contact between the two cells [43]. This effect is mediated through the release of numerous EVs containing galectin-10 by eosinophils [43]. Similarly, the anti-inflammatory effects of eosinophil-derived EVs have also been demonstrated in functional dyspepsia (FD). As a form of functional gastrointestinal disorders, FD is generally regarded as a non-organic disease. However, recent studies revealed organic alterations in functional gastrointestinal disorders, such as impaired epithelial barrier function [67]. In fact, the descending part of the duodenum in patients with FD is often accompanied by mild inflammation, which is thought to be linked to elevated mucosal permeability [68]. Furthermore, research indicated an association between immune dysregulation and FD [69]. The pathogenesis of FD is often accompanied by activation of eosinophils and mast cells [70]. Wang et al. discovered that EVs derived from eosinophils and mast cells alleviate FD symptoms [44]. Specifically, the LncRNA NEAT1 within these vesicles targets miRNA-211-5p, thereby upregulating glial cell line-derived neurotrophic factor expression and promoting duodenal mucosal integrity [44].

The gastrointestinal mucosa consists of delicate regenerative epithelium that is vulnerable to injury from ionizing radiation [71]. This renders radiation enteritis one of the most prevalent and severe complications in patients receiving abdominal or pelvic radiotherapy [71]. Ionizing radiation activates diverse cellular signaling pathways, leading to the expression and activation of pro-inflammatory and pro-fibrotic cytokines, vascular damage, and initiation of the coagulation cascade [72, 73]. Therapeutic options for radiation enteritis are limited. A study by Zhou et al. found that T cell-derived EVs can hydrolyze intracellular and extracellular cGAMP via the surface enzyme ENPP1, inhibit the radiation-activated cGAS-STING pathway, and thereby alleviate radiation enteritis [45]. Similarly, Saha et al. also demonstrated the beneficial effects of immune cell-derived EVs [46]. The research team discovered that WNTs packaged within macrophage-derived EVs could rescue intestinal stem cells and enhance survival rates in mice undergoing radiation injury [46].

*Helicobacter pylori* (*H. pylori*) is a Gram-negative bacterium that colonizes the human gastric mucosa [74]. H. pylori-associated chronic gastritis is one of the most common gastrointestinal diseases. For decades, complete eradication of this pathogen has posed a persistent dilemma for both clinicians and patients. One primary function of immune cells is to eliminate exogenous pathogens. However, during disease progression, *H. pylori*, immune cells, and gastric epithelial cells mutually maintain a delicate homeostatic balance [75]. The underlying mechanism is that *H. pylori* reshapes host immune responses to avoid clearance by the body through a series of strategies, such as cholesterol glycosylation and evasion of Toll-like receptor recognition [76, 77]. Regrettably, the immune privilege induced by H. pylori-driven immune suppression is insufficient to offset the potent pro-inflammatory activities mediated by pathogen-associated molecular patterns (PAMPs) and damage-associated molecular patterns (DAMPs) from injured epithelial cells [78]. By acting on pattern recognition receptors, PAMPs and DAMPs activate downstream signaling cascades, recruit inflammatory cells, predominantly neutrophils, and prompt the massive production of inflammatory cytokines, thereby triggering inflammatory responses [49, 79]. Nevertheless, recent studies have demonstrated that pro-inflammatory EVs derived from immune cells and their cargo do not always adversely affect disease outcomes in chronic gastritis [47]. In fact, from another perspective, inflammation constitutes an indispensable complex pathological process that maintains organismic homeostasis [80]. During *H. pylori* infection, inflammatory responses, especially those mediating pathogen clearance, suppress the survival of *H. pylori* — the primary causative agent of chronic gastritis. This mechanism, to a certain degree, protects the host against infection-related damage. The miRNA-155 (a widely reported pro-inflammatory miRNA) carried by macrophage EVs can promote the expression of inflammatory cytokines and CD40, CD63, CD81, and MCH-I in H. pylori-infected macrophages [47]. This process regulates the inflammatory response of target cells, promoting their inhibition or killing of the bacterium and thereby preventing gastritis [47]. Similarly, from another perspective, EVs derived from anti-inflammatory immune cells and the activation of M2 macrophages are not always beneficial to the prognosis of patients with gastrointestinal inflammation. It has been reported that chronic inflammation drives the synthesis of miRNA-93-5p in G-MDSCs, which further mediates the differentiation of M-MDSCs into M2 macrophages through EVs [81]. Excessive M2 macrophage activation promotes the transition from inflammation to tumor [81].

Admittedly, numerous studies have explored the biological functions of macrophage- and neutrophil-derived EVs in colitis. Nevertheless, investigations into EVs derived from other immune cells in colitis, as well as the roles of immune cell-derived EVs in gastrointestinal inflammation beyond colitis, remain scarce, leaving substantial gaps in this research field. Furthermore, within the context of gastrointestinal inflammation, the pro-inflammatory and anti-inflammatory roles of immune cells and their EVs require an in-depth understanding and dialectical consideration. On the one hand, attention should be paid to the heterogeneity of immune cells. On the other hand, we must carefully weigh the merits and limitations of immune cell-derived EVs with respect to disease progression and patient prognosis. Lastly, EV-mediated crosstalk between immune cells during gastrointestinal inflammation remains poorly understood in the scientific community. This field urgently requires more comprehensive, in-depth investigations to establish a comprehensive regulatory network of immune cells, thereby better elucidating the biological functions of these cells and their EVs in gastrointestinal inflammation.

## Therapeutic potential of EVs

**FIGURE 3:**
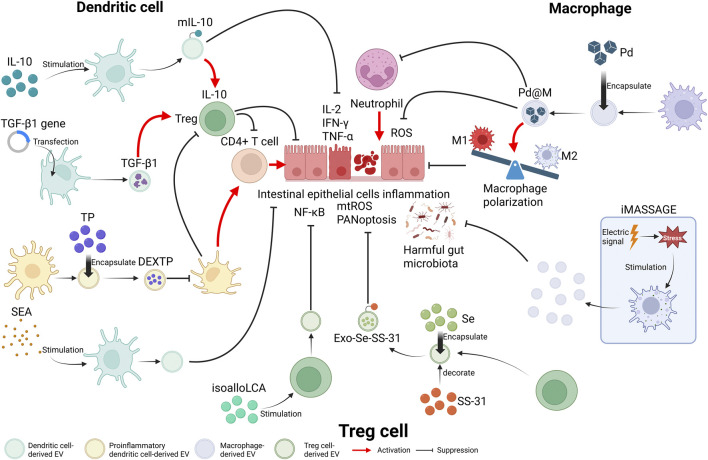
Application of modified immune cell EVs for enhancing gastrointestinal inflammation treatment. This figure shows that modified EVs derived from dendritic cells, macrophages, and regulatory T cells can alleviate gastrointestinal inflammation. Modification strategies can be divided into functional modification and quantity modification. Dendritic cells can achieve indirect functional modification of their derived EVs via stimulation with IL-10, SEA, or TGF-β1 overexpression, and perform direct functional modification by encapsulating TP inside these EVs. Macrophages can perform direct functional modification by encapsulating Pd inside their derived EVs, and achieve quantity modification of these EVs via electromechanical stimulation. Regulatory T cells can achieve indirect functional modification of their derived EVs via isoalloLCA stimulation, and perform direct functional modification by encapsulating Se and decorating EV membranes with SS-31. Modified EVs can act on intestinal epithelial cells or other immune cells, thereby alleviating inflammation in intestinal epithelial cells directly or indirectly. Created with BioRender.com.

Despite advances in biologics, including various monoclonal antibodies, drugs currently used to treat gastrointestinal inflammation still exhibit low bioavailability and poor targeting [82]. This is reflected in their limited accumulation at inflamed sites, along with numerous severe adverse reactions caused by systemic immunosuppression [82]. To address this issue, it is essential to develop new therapeutics based on biological nanotechnology. Advanced nanomedicines are expected to feature high reliability and potent targeting capability. They must also resist the harsh gastrointestinal environment and achieve efficient drug delivery to inflamed regions [83]. Such formulations can raise local drug concentrations, improve therapeutic outcomes, and reduce systemic side effects [83]. Sun et al. constructed a nanotherapeutic formulation in which therapeutic agents are encapsulated within macrophage membranes, enabling efficient targeting of inflamed sites and modulating macrophage polarization [84]. Leveraging the intrinsic chemotactic traits and excellent biocompatibility of macrophage membranes, this work provides important references for the design of nanomedicines against inflammatory disorders [84]. As key mediators of intercellular signaling, immune cell-derived EVs participate in a wide range of biological processes. They exhibit favorable chemotactic properties, superior biocompatibility, and stable cargo-carrying capacity. Given that their membrane structure is highly similar to that of cell membranes, EVs can largely evade immune surveillance and clearance. Accordingly, EV-based delivery systems hold great potential to improve targeted drug delivery and boost therapeutic efficacy for clinical treatment, showing promising application prospects [85]. In the following section, we elaborate on the therapeutic potential of immune cell-derived EVs in gastrointestinal inflammation (Table 2; Figure 3).

**TABLE 2:** Application of modified immune cell EVs for enhancing gastrointestinal inflammation treatment.

EVs modification strategy	Source of EVs	Specific modification methods	Experimental model	Type of delivery	Therapeutic target	Main effects	References
Indirect functional modification	DCs	IL-10 stimulation	TNBS-induced rat colitis model	Intraperitoneal injection	Th1-type inflammation, colonic lamina propria Tregs	Downregulate IL-2, IFN-γ, and TNF-α mRNA expression; markedly increase Treg count in colonic lamina propria	[86]
​	​	SEA stimulation	DSS-induced mice colitis model	Intraperitoneal injection	Inflammatory cytokine expression	Downregulate pro-inflammatory cytokines (TNF-α, IFN-γ, IL-17A, IL-12, IL-22); upregulate anti-inflammatory cytokine (TGF-β)	[87]
​	​	Overexpression of TGF-β	DSS-induced mice colitis model	Tail vein injection	Th17/Treg balance	Significantly induced Tregs and attenuated Th17-mediated inflammatory response	[88]
​	Tregs	IsoalloLCA stimulation	DSS-induced mice colitis model	Tail vein injection	NF-κB signaling pathway	More effectively alleviate colitis by inhibiting NF-κB-associated inflammation	[89]
Direct functional modification	Macrophages	Import cubic palladium	DSS-induced mice colitis model	Tail vein injection	Oxidative stress, macrophage polarization, neutrophil activity	Effectively clear ROS, regulate the polarization of macrophages, and reduce the infiltration and recruitment of neutrophils	[90]
​	DCs	Import TP	TNBS-induced mice colitis model	Tail vein injection	DCs and DC-mediated CD4^+^ T/Treg immune balance	Inhibit DC activation and induce DC apoptosis, which further induces T-cell immunosuppression	[91]
​	Tregs	Import L-selenocystine Conjugate SS-31 tetrapeptide	DSS-induced mice colitis model	Tail vein injection	Mitochondrial oxidative stress, PANoptosis	More effectively and precisely prevent intestinal inflammation from PANoptosis by blocking mitochondrial oxidative stress	[92]
Quantitative modification	Macrophages	Electromechanical stimulation	DSS-induced mice colitis model	Intraperitoneal implantation	Gut microbiota and intestinal inflammation	Correct intestinal microbiota imbalance and attenuate colitis	[93]

*EVs*, extracellular vesicles; *DCs*, dendritic cells; *IL*, interleukin; *IFN*, interferon; *TNF*, tumor necrosis factor, *SEA* S. japonicum soluble eggs antigen, *DSS*, dextran sulfate sodium; *TNBS*, 2,4,6-trinitrobenzenesulfonic acid, *LPS*, lipopolysaccharide; *TGF*, transforming growth factor, *Tregs* regulatory T cells, *Th17* type 17 helper T, *Th1* type 1 helper T, *ROS*, reactive oxygen species; *TP*, triptolide, *IsoalloLCA*, isoallolithocholic acid.

### Functional modification

In the field of inflammatory therapy using immune cell-derived EVs, functional modification of these vesicles has become the most common strategy for developing new nanotherapeutics [94, 95]. Multiple studies have confirmed that functionally modified EVs enable more effective regulation of immune responses and promotion of tissue repair, leading to improved therapeutic efficacy. Methods for functionally modifying EVs can broadly be categorized into two approaches: indirect modification via treatment of parental cells to naturally alter their secreted EVs, and direct modification of isolated EVs.

### Indirect functional modification

It has been reported that when macrophages are infected with drug-resistant parasites, the proteome of macrophage-derived EVs undergoes substantial changes that further regulate immune responses [96]. This finding indicates that immune cells can alter the components of their EVs to exert corresponding functions in response to external stimuli. In the treatment of gastrointestinal inflammation, dendritic cell-derived EVs are most commonly modified to enhance therapeutic efficacy. Yang et al. treated dendritic cells with IL-10; intraperitoneal injection of these modified EVs markedly reduced the severity of TNBS-induced rat colitis, downregulated mRNA expression of IL-2, IFN-γ, and TNF-α in colonic tissue, and upregulated IL-10 mRNA expression in colonic Tregs [86]. Wang et al. treated dendritic cells with Schistosoma japonicum soluble egg antigens and found that EVs from treated dendritic cells more effectively ameliorated DSS-induced acute colitis in mice [87]. Cai et al. directly overexpressed TGF-β in dendritic cells to generate immunosuppression-associated EVs [88]. Functionally modified EVs significantly induced Tregs and attenuated Th17-mediated inflammatory responses [88]. Besides dendritic cells, indirect modification of EVs from Tregs may also achieve better therapeutic effects. In a recent study, researchers stimulated Tregs with isoallolithocholic acid. They found that EVs from stimulated Tregs could more effectively suppress NF-κB-related inflammation in intestinal epithelial cells, thereby alleviating inflammatory bowel disease [89]. Indirect functional modification of EVs features relatively simple and mild preparation procedures. This approach only requires treatment of parental cells to alter the properties of their derived EVs. Nevertheless, it remains challenging to identify the key functional molecules in EVs and enrich them efficiently, since the packaging of these molecules into EVs depends entirely on the intrinsic biological processes of parental cells.

#### Direct functional modification

Compared with indirect modification via treatment of parental cells, advances in nanobiotechnology have enabled direct modification of EVs. Momen-Heravi et al. discovered that miRNA-155 inhibitors, delivered to macrophages via B-cell-derived EVs, could significantly reduce inflammatory cytokine expression in macrophages and exhibit lower cytotoxicity [97]. This study suggests that loading anti-inflammatory agents into immune cell-derived EVs can more effectively alleviate inflammation, offering insights for modifying vesicle cargos to treat gastrointestinal inflammation. Regarding gastrointestinal inflammation therapy, Cheng et al. engineered a biomimetic nanotherapeutic for ulcerative colitis by incorporating cubic palladium into macrophage-derived EVs [90]. This drug exhibited favorable targeting and biocompatibility, effectively scavenging reactive oxygen species, inhibiting glycolysis, modulating macrophage polarization, and reducing neutrophil infiltration and recruitment [90]. Triptolide shows therapeutic potential for ulcerative colitis, though its multi-organ toxicity requires resolution [98, 99]. Dendritic cells serve as the primary target for triptolide-induced immunosuppression, and dendritic cell-derived EVs can selectively delivery to dendritic cells *in vivo* [100, 101]. Therefore, Rao et al. encapsulated triptolide within dendritic cell-derived EVs to achieve targeted triptolide delivery, thereby mitigating colonic inflammation while reducing therapeutic toxicity [91]. All of these studies used biological nanotechnology to modify the cargo within EVs to regulate immune responses. Furthermore, EV membranes can be modified to enhance targeting and therapeutic efficacy. In Gong’s study, researchers not only loaded L-selenocystine into Treg-derived EVs to enhance anti-inflammatory activity but also modified the EV membrane surface with the mitochondrial-targeting tetrapeptide SS-31 to improve targeting [92]. The modified EVs effectively suppressed mitochondrial oxidative stress and PANoptosis, thereby effectively alleviating intestinal inflammation [92]. In contrast to indirect EV modification, direct modification allows targeted loading of predetermined key effectors into EVs. This approach is independent of cells' intrinsic biological processes and involves fewer variables and uncertainties. Nevertheless, direct modification of EVs involves relatively complicated preparation procedures. Moreover, certain loading methods, such as ultrasonication, electroporation, or extrusion, may damage the vesicle membrane, alter the biological properties of EVs, and impair their functions.

### Quantity modification

Even more encouraging is that, nowadays, beyond functional modification of EVs, advances in biological nanotechnology and cross-disciplinary integration have made it possible to control the quantity of therapeutic EVs. That is, the therapeutic potential of EVs can be further explored through quantitative changes. The most representative example is a micro wireless bioelectronic system developed by Wan et al, named “iMASSAGE” [93]. This system uses a bioelectronic controller and a hydrogel to deliver electromechanical stimulation to embedded macrophages in the body, thereby generating therapeutic EVs that increase up to 20-fold over baseline to correct gut microbiota dysbiosis and ameliorate colitis [93]. This *in vivo* controllable EV generation system boosts the production of therapeutic EVs and extends their duration of action. It not only marks a landmark progress in the treatment of gastrointestinal inflammation, but also serves as a promising therapeutic platform for other diseases. However, further validation of its safety and efficacy in humans is still required before this system can be fully applied in clinical practice. In the future, researchers may select parental cells with superior anti-inflammatory effects (e.g., Tregs) or combine this strategy with EV functional modification to enhance their therapeutic outcomes.

## Discussion

Gastrointestinal inflammation occupies a significant position among digestive system diseases, with current treatment options remaining limited. The incidence and prevalence of chronic gastrointestinal inflammation, represented by inflammatory bowel disease, have risen annually. This not only severely impacts public health but also imposes a huge burden upon national healthcare systems. Immune cells play significant roles in the pathophysiology of gastrointestinal inflammation. Furthermore, as research has deepened, the role of immune cells in influencing gastrointestinal inflammation through EVs has gradually gained recognition within the scientific community.

During gastrointestinal inflammation, EVs derived from immune cells can regulate the digestive tract immune system while also directly acting on gastrointestinal epithelial cells to contribute to tissue damage or repair. Investigating the role of immune cell-derived EVs in gastrointestinal inflammation is of great significance. On the one hand, the research mentioned above has identified molecular targets of gastrointestinal inflammation, offering novel insights into elucidating its pathogenesis. This further reveals the mechanisms of such inflammation. On the other hand, such studies have also revealed the immune-regulatory roles mediated by EVs, providing theoretical support for using EV-mediated cell crosstalk to alleviate gastrointestinal inflammation. This provides novel ideas and targets for its treatment. At present, most studies on the roles of immune cell-derived EVs in gastrointestinal inflammation focus mainly on macrophages and neutrophils, and the related research is largely limited to colonic inflammation. In future work, the mechanisms whereby other immune cells regulate a broader spectrum of gastrointestinal inflammation via EVs remain to be elucidated.

In the treatment of gastrointestinal inflammation, the development of novel, highly targeted, and reliable therapeutics based on biological nanotechnology is imperative. EVs have garnered significant interest from researchers due to their excellent biocompatibility, tropism, and loading capacity. Explorations into the use of immune cell-derived EVs to treat gastrointestinal inflammation include functional modification and quantitative control. It must be noted that although there are currently sufficient studies that have explored the therapeutic potential of EVs, these studies are still in the nascent stages. In fact, there are still considerable challenges in developing EVs into next-generation nanotherapeutic drugs and in their true application in clinical practice.

Firstly, the composition of EVs is complex, and the mechanisms by which immune cell-derived EVs act in gastrointestinal inflammation remain incompletely elucidated. Indirect functional modification of these vesicles through stimulation of parent cells complicates the identification of the substances that truly exert therapeutic effects, leaving room for improvement in both therapeutic efficacy and safety. Secondly, current methods for directly functional modifying EVs remain limited. Conventional physical and chemical modification techniques suffer from low efficiency and may partially damage EV membranes, leading to vesicle aggregation or denaturation. Accordingly, improving modification efficiency while maintaining the structural integrity and functional activity of EVs remains a critical bottleneck that requires urgent breakthroughs. Moreover, despite the diverse biological functions of EVs, their large-scale extraction, purification, quantification, and preservation remain major challenges that need to be resolved. To address this problem, it is essential to establish standard quality-control protocols and comprehensive processing workflows to maintain the morphology, structural integrity, and functional activity of therapeutic EVs. Finally, although the therapeutic potential of EVs has been validated in animal models, their efficacy and safety in human subjects remain unclear. Issues concerning effective EV delivery in humans and the selection of optimal dosing regimens remain pressing challenges that call for intensive research in related fields. Moving forward, concerted efforts are required to address the aforementioned problems. In addition, we should integrate existing strategies to improve the therapeutic performance of EVs, such as functional modification and quantity control. It is also necessary to fully explore the therapeutic potential of these vesicles from multiple perspectives to achieve better treatment outcomes.
